# The impact of Bayesian optimization on feature selection

**DOI:** 10.1038/s41598-024-54515-w

**Published:** 2024-02-17

**Authors:** Kaixin Yang, Long Liu, Yalu Wen

**Affiliations:** 1 Department of Health Statistics, School of Public Health, Shanxi Medical University, No 56 Xinjian South Road, Yingze District, Taiyuan, Shanxi China; 2 Department of Statistics, University of Auckland, 38 Princes Street, Auckland Central, Auckland, 1010 New Zealand

**Keywords:** Biomarkers, Computational biology and bioinformatics

## Abstract

Feature selection is an indispensable step for the analysis of high-dimensional molecular data. Despite its importance, consensus is lacking on how to choose the most appropriate feature selection methods, especially when the performance of the feature selection methods itself depends on hyper-parameters. Bayesian optimization has demonstrated its advantages in automatically configuring the settings of hyper-parameters for various models. However, it remains unclear whether Bayesian optimization can benefit feature selection methods. In this research, we conducted extensive simulation studies to compare the performance of various feature selection methods, with a particular focus on the impact of Bayesian optimization on those where hyper-parameters tuning is needed. We further utilized the gene expression data obtained from the Alzheimer's Disease Neuroimaging Initiative to predict various brain imaging-related phenotypes, where various feature selection methods were employed to mine the data. We found through simulation studies that feature selection methods with hyper-parameters tuned using Bayesian optimization often yield better recall rates, and the analysis of transcriptomic data further revealed that Bayesian optimization-guided feature selection can improve the accuracy of disease risk prediction models. In conclusion, Bayesian optimization can facilitate feature selection methods when hyper-parameter tuning is needed and has the potential to substantially benefit downstream tasks.

## Introduction

The significance of risk prediction for complex diseases originates from its capacity to enable personalized medicine strategies by accurately assessing an individual's susceptibility to diverse diseases^1^. Emerging high-dimensional molecular data has immense potential to offer prospective insights into the underlying causes of complex diseases, thereby greatly facilitating risk prediction. However, the excessive amount of irrelevant and redundant features in the high-dimensional molecular data can not only lead to over-fitting and unrobust models but also significantly increase computational costs^2^. Therefore, feature selection becomes an indispensable step when building prediction models with high-dimensional molecular data^3^.

Existing feature selection methods can be broadly classified into three categories^4^. Filter-based methods, such as sure independence screening (SIS)^5^ and minimum redundancy maximum relevance (MRMR)^6^, first perform feature selection on the datasets, and then train the predictive model. The feature selection process is independent of the subsequent predictive model. They are generally easy to implement, but their selected features cannot guarantee to achieve the best performance for the subsequent tasks. Wrapper-based methods [e.g., recursive feature elimination (RFE)^7^] directly evaluate the performance of the final predictive model to determine the quality of the feature subset. While they take the subsequent analyses into consideration, wrapper-based methods tend to be computationally intensive and can lead to over-fitting. Embedded-based methods, such as least absolute shrinkage and selection operator (Lasso)^8^, elastic net (Enet)^9^, and extreme gradient boosting (XGBoost)^10,11^, incorporate the feature selection process into the objective functions of prediction modeling. For example, Lasso adds an L_1_ penalty term into the traditional loss function to simultaneously optimize the prediction accuracy and select important predictors. Despite the wide applications, the performance of many embedded-based methods depend on the values of hyper-parameters^12^. For instance, Lasso requires the specification of a tuning parameter to determine the sparsity of the model^13^. When XGBoost is employed for feature selection, hyper-parameters (e.g., learning rate that makes trade-off between model generalization and convergence rate, and maximum depth that balances between under and over-fitting) can significantly influence its stability and efficiency^14^.

Hyper-parameter tuning can be an extremely challenging task. Traditional manual tuning, such as optimizing the learning rate and batch size of the neural network, is not only computationally demanding, but also unlikely to provide hyper-parameters that can guarantee the best performance^15^. Recently, hyper-parameter optimization that aims at automating the process has obtained much attention in various fields^16–19^. For example, within the field of brain disorders, hyper-parameter optimization has been used to facilitate image processing^20,21^ and disease classification^22^. Existing widely used hyper-parameter optimization techniques encompass grid search^23^, random search^24^, and Bayesian optimization^25^. However, both grid search and random search are not directly applicable for the analysis of high-dimensional data. Grid search evaluates all feasible hyper-parameter combinations and thus its computation can be extremely demanding for high-dimensional data. Random search avoids the exhaustive evaluation by using a sampling strategy. However, the sampling process does not leverage the information from prior evaluations, which can lead to sub-optimal hyper-parameter combinations. Unlike grid and random search, Bayesian optimization uses probabilistic models to guide the search, enabling adaptive sampling of hyper-parameters and focusing on promising regions. Therefore, it reduces the computational cost, enhances stability, and has the potential to provide an optimal hyper-parameter configuration^25^.

While Bayesian optimization has demonstrated its advantages in many domains (e.g., resource allocation of web-pages^26,27^, gaming^28^ and sensor networks^29^), its role for optimizing feature selection remains under-investigated, especially for high-dimensional data. Our main contribution in this research is to provide insights into the utility and feasibility of Bayesian optimization in dimension reduction methods, especially when it is applied to high-dimensional molecular data. We hypothesized that incorporating Bayesian optimization into feature selection for the analysis of high-dimensional molecular data can improve the robustness and accuracy of existing methods whose performance can be affected by hyper-parameters. We first conducted extensive simulation studies to evaluate the impact of Bayesian optimization on existing widely used methods for the analysis of high-dimensional data, and then analyzed gene expression data obtained from Alzheimer's Disease Neuroimaging Initiative (ADNI) to gauge the impact of Bayesian optimization. Finally, we provided some practical suggestions for using Bayesian optimization in the analysis of big molecular data.

## Materials and methods

In the following sections, we first provided the technical details of all the analytical methods that we have considered, and then presented the settings of simulation studies. Finally, we detailed the procedure adopted in the analysis of high-dimensional gene expression data obtained from ADNI.

### Technical details

As we predominantly focused on the impact of Bayesian optimization on feature selection, we first provided details of Bayesian optimization. We then presented commonly used feature selection methods, including those with and without hyper-parameters.

#### Bayesian optimization

Bayesian optimization^18^ aims at finding the combinations of hyper-parameters that maximizes the performance of the original problem. It defines its objective function in the same fashion as the original problem, and iteratively estimates the posterior distribution of the objective function as well as the subspaces of the hyper-parameters that are likely to achieve the optimized objective function. To be specific, for each iteration, Bayesian optimization first uses the uniform distribution to sample hyper-parameters from a given range and adopts the Gaussian process to estimate the posterior distribution of the objective function for each sampled hyper-parameter combination. It then identifies the region of hyper-parameters that are likely to optimize the objective function using the acquisition function, which balances between the posterior mean ($\mu \left(x\right))$ and variance ($\sigma \left(x\right))$ of the objective function using the tuning parameter $\kappa$, it can be expressed as Eq. (1):

1 $$AC\left(x\right)=\mu \left(x\right)+\kappa \sigma \left(x\right)$$

By utilizing the acquisition function to identify the optimal subspace of hyper-parameters, Bayesian optimization can avoid the region that achieves the best value of the objective function but has a considerable large variance. By iteratively estimating posterior distribution of the objective function and adaptively updating the subspace of hyper-parameters, Bayesian optimization can leverage the information from prior evaluations and only focus on promising subspaces, which not only simplifies the hyper-parameter searching process, but also improves the stability and tends to provide an optimal hyper-parameter configurations^25^.

#### Feature selection methods

We considered embedded-based methods (i.e., Lasso, Enet, and XGBoost) that require hyper-parameter tuning as well as filter-based (i.e., SIS and MRMR) and wrapper-based methods (i.e., sPLSda) that do not involve hyper-parameters for comparison purposes. Lasso, Enet and XGBoost exhibit greater versatility and have been experimentally applied in research on brain disorders and gene expression^30,31^. SIS, MRMR, and sPLSda are also widely utilized for feature selection in gene expression data^32–34^.

Lasso^8^ and Enet^9^ are penalized regression models with objective function of the form:

2 $${f}_{w}\left(x\right)+\lambda \rho {\Vert w\Vert }_{1}+\frac{\lambda \left(1-\rho \right)}{2}{\Vert w\Vert }_{2}^{2},$$

where ${f}_{w}\left(x\right)$ is the loss function (e.g., mean square error) and $\lambda$ is a tuning parameter controlling the sparsity of the model. $\rho$ controls the relative contributions of the two penalties, where *L*_*1*_ penalty term (i.e., ${\Vert w\Vert }_{1}$) encourages sparsity with coefficients of less important factors being shrunk to zero and *L*_*2*_ penalty term (i.e., ${\Vert w\Vert }_{2}^{2}$) encourages shrinkage to reduce the impact of over-fitting and multi-collinearity. Lasso sets $\rho =1$ and Enet sets $0<\rho <1$. Both Lasso and Enet involve a hyper-parameter $\lambda$, which controls the sparsity of the model and normally is chosen in accordance with information criteria [e.g., Akaike information criterion (AIC)] and cross-validation (CV). In this research, we explored the benefits of using Bayesian optimization on the hyper-parameter $\lambda$ and $\rho$. We utilized Bayesian optimization to adjust the values of $\lambda$ for Lasso and $\rho$ and $\lambda$ for Enet, both of which fall within the range of (0, 1).

XGBoost^11^ is built based on the decision tree with an objective function of Eq. (3):

3 $${Obj}^{*}\approx \gamma T-\frac{1}{2}\sum_{j=1}^{T}\frac{{G}_{j}^{2}}{{H}_{j}+\lambda }$$

where $\gamma$ and $\lambda$ can control the strength of the penalty. $T$ is the total number of leaf nodes, and ${G}_{j}$, ${H}_{j}$ represent the sum of the first and second order gradients of all samples of the $j$-th node, respectively. XGBoost can be used as a variable selection tool as it can gauge the importance of each feature via the ‘gain’, which measures the improvement of the objective function once the feature is split and is defined as Eq. (4):

4 $$Gain= \frac{1}{2}\cdot \left[\frac{{G}_{L}^{2}}{{H}_{L}+\lambda }+\frac{{G}_{R}^{2}}{{H}_{R}+\lambda }-\frac{{\left({G}_{L}+{G}_{R}\right)}^{2}}{{H}_{L}+{H}_{R}+\lambda }\right]-\gamma$$

where subscripts *L* and *R* respectively denoting the left and right tree after splitting at the node. A larger value of the ‘gain’ signifies a superior split, highlighting the increased importance of the feature. In this study, we explored the advantages of Bayesian optimization on the optimization of hyper-parameters that are listed in Supplementary Table S1.

SIS^5^ is a filter-based feature selection method that ranks the importance of features by evaluating their correlations with the target. It first computes the association between each feature and the target, and then selects a subset of features according to the rank of their correlation values based on a pre-specified user-defined number of features.

MRMR^6^ ranks the features on the basis of their mutual information with the target and their mutual information with other, which can be expressed as:

5 $$MRMR\left(i\right)=MI\left(y,{x}_{i}\right)-\frac{1}{S}\cdot {\sum }MI\left({x}_{i},{x}_{j}\right)$$

where $MI\left(\cdot ,\cdot \right)$ represents mutual information. MRMR aims at selecting a subset of features that maximizes the correlation with the target while minimizing the redundancy among features. Similar to SIS, it selects the top features with the highest MRMR score for downstream analysis.

sPLSda^35^ predominantly aims at converting the original predictor into a small number of orthogonal latent variables that are constructed with a small set of inputs and maximizes the covariance between input and output. It uses the objective function of the partial least square discriminant analysis to form the latent variables and imposes a penalty term to allow that latent variables are constructed with a selected number of features in the original space. It defines its objective function as:

6 $$\underset{{\Vert \alpha \Vert }^{2}=1, {\Vert \beta \Vert }^{2}=1}{{\text{argmax}}}co{v}^{2}\left(T, H\right)\mathrm{ with }T=X\alpha \mathrm{\,and\, }H=Y\beta ,$$

where $\alpha$ is the penalty term. As sPLSda is designed for classification problem, we categorized the response into 50 categories following Bommert et al.^36^.

### Simulation studies

To evaluating whether the Bayesian-optimized feature selection method could yield more stable and reliable feature selection, we conducted simulation studies. We simulated the covariates ${{\varvec{X}}}_{{\varvec{i}}}={\left( {X}_{i1}, {X}_{i2},\cdots , {X}_{ip}\right)}^{{\text{T}}}$ of the *i*-th individual as ${{\varvec{X}}}_{{\varvec{i}}}\boldsymbol{ }\sim \boldsymbol{ }N(0,{{\varvec{I}}}_{{\varvec{p}}})$, where ${{\varvec{I}}}_{{\varvec{p}}}$ is an identify matrix and *p* is set to be 5000. We considered both linear and non-linear models. For the linear effects, the continuous outcomes were simulated as:

7 $${Y}_{i}={{\varvec{X}}}_{{\varvec{i}}}{\varvec{w}}+{\varepsilon }_{i},$$

where ${{\varvec{X}}}_{{\varvec{i}}}={\left( {X}_{i1}, {X}_{i2},\cdots , {X}_{i{p}_{0}}\right)}^{{\text{T}}}$ is the causal covariate**,** ${\varvec{w}}=({w}_{1},{w}_{2},\cdots , {w}_{{p}_{0}})$ is their corresponding effect and ${\varepsilon }_{i} \sim N(\mathrm{0,1})$. Following the same settings used in Fan and Lv^5^, we set the ${w}_{i}$ as:

8 $${w}_{i}={\left(-1\right)}^{{U}_{i}}\cdot \left(\frac{5\cdot {\text{log}}\left(n\right)}{\sqrt{n}}+\left|{Z}_{i}\right|\right),$$

where ${U}_{i}\sim Ber(0.5)$ and ${Z}_{i}\sim N\left(\mathrm{0,1}\right).$ For the non-linear model, the continuous outcomes were simulated as:

9 $${Y}_{i}={\sum }_{j=1}^{{p}_{1} }\mathit{sin}\left({X}_{ij}\right)+1.8\times {\sum }_{j={p}_{0}-{p}_{1}+1}^{{p}_{0} }\mathit{cos}\left({X}_{ij}\right)+ {\varepsilon }_{i},$$

where ${p}_{1}=\frac{{p}_{0}}{2}$, and ${\varepsilon }_{i}\sim N\left(\mathrm{0,0.1}\right)$. To consider binary outcomes under both linear and non-linear effect models, we generated ${{Y}_{i}}$ = $$\left\{\begin{array}{c}0, {Y}_{i} < {Y}_{mid}\\ 1, {Y}_{i} \ge {Y}_{mid}\end{array}\right.$$ and ${Y}_{mid}$ is the median of the set ${Y}_{i}$. We set the total sample size to be 1000, and gradually increased the number of causal features from 100 to 1000 (i.e., ${p}_{0}=\left(100, 200, 500, 1000\right)$). We repeated each simulation setting 20 times.

We implemented Bayesian optimization on XGBoost (denoted as BO_XGBoost), Lasso (denoted as BO_Lasso), and Enet (denoted as BO_Enet), where hyper-parameter tuning is needed. We set the number of iterations to be 100 for Bayesian optimization. We also considered the above three methods, where the hyper-parameters were set based on their default settings of the corresponding R packages (i.e., xgboost^37^, glmnet^38^ and msaenet^39^). To make fair comparisons, we included additional three widely used feature selection methods (i.e., SIS, sPLSda, MRMR), where hyper-parameters are not involved. For each method, we varied the pre-specified number of selected features from 100 to 1000 (i.e., ${n}_{s}=(100, 200, 500, 1000))$. We used the recall rate to evaluate the performance of each feature selection method.

### The analysis of gene expression data from ADNI

ADNI^40,41^, including ADNI 1, ADNI 2, ADNIGO, and ADNI 3, is a comprehensive longitudinal study aimed at identifying biomarkers associated with Alzheimer's disease (AD) and advancing its clinical diagnosis. It furnishes an extensive array of imaging data, encompassing MRI and PET scans, alongside cerebral spinal fluid, and blood biomarkers. In addition, it also includes several clinical and neuropsychological measures obtained from three distinct groups: healthy controls, individuals with mild cognitive impairment, and those diagnosed with AD. This measures collection spans a duration of 3 years, with an additional 6 years of data acquisition facilitated by the ADNI-GO and ADNI-2 projects^42^. The details are presented in Wyman et al.^43^.

We focused on brain imaging traits from ANDI studies. These traits include subcortical volumes (hippocampus, accumbens, amygdala, caudate, pallidum, putamen, thalamus), the volumes of gray matter, white matter and brainstem + 4th ventricle from T1 structural brain MRI, and the volume of white matter hyperintensities from T2-weighted brain MRI. We have normalized the phenotype data, and the sample sizes for each phenotype in ADNI studies are summarized in Table 1. Demographic information is summarized in Supplementary Table S2.

**Table 1:** The sample size and distributions of eleven brain imaging traits in the Alzheimer’s Disease Neuroimaging Initiative study.

Phenotype	Sample size	Mean ± SD (${{\text{mm}}}^{3}$)	Q1	Median	Q3
Hippocampus	595	7024 ± 1102.958	6270	7080	7790
Accumbens	493	957 ± 173.643	834	947	1061
Amygdala	493	2709 ± 470.905	2407	2704	3034
Caudate	493	6915 ± 1008.494	6208.5	6806	7373.5
Pallidum	493	3026 ± 397.589	2777	3008	3244
Putamen	493	9425 ± 1195.311	8644	9369	10,060
Thalamus	493	12,371 ± 1353.091	11,460	12,243	13,190
Gray matter	434	598,496 ± 55,963.684	559,538.5	598,675.5	634,992.25
White matter	434	468,448 ± 62,876.207	424,171.75	467,431	509,600.25
Brainstem + 4th ventricle	305	20,744 ± 2298.567	1541.7725	3454.8	8039.4
${\mathrm{White matter hyperintensity}}^{{\text{a}}}$	434	6915 ± 10,391.908	19,152.75	20,496.5	22,291.5

^*a*^White matter hyperintensity, logarithm to base 10 was performed to make it to be approximately normal distribution.

Gene expression data were derived from blood samples collected from the 811 participants in the ADNI WGS cohort. Analysis was conducted using the Affymetrix Human Genome U219 Array (Affymetrix, Santa Clara, CA). We utilized the data after quality control from K.N. et al.^44^, and no additional quality control steps were applied. For our analysis, we extracted 49,386 gene expression profiles that pass the quality control criteria for subsequent modeling.

For ADNI data, we genuinely lack knowledge on which genes are related to AD, and thus we rely on the predictive performance to determine whether we have identified AD risk factors and achieved a robust and accurate AD risk prediction model. We split the data into a 70% training set and a 30% testing set. We employed all feature selection methods in simulation studies to select a pre-specified number of features from the training data, and then fed these features to the downstream prediction tasks, where support vector machine (SVM), Lasso, random forest (RF) and gradient boosting machine (GBM) are used to build prediction models. We evaluated the prediction performance based on testing data, where Pearson correlations and mean square errors are calculated. We repeated this process 100 times to avoid chance finding.

## Result

### Result from simulation studies

**Figure 1:**
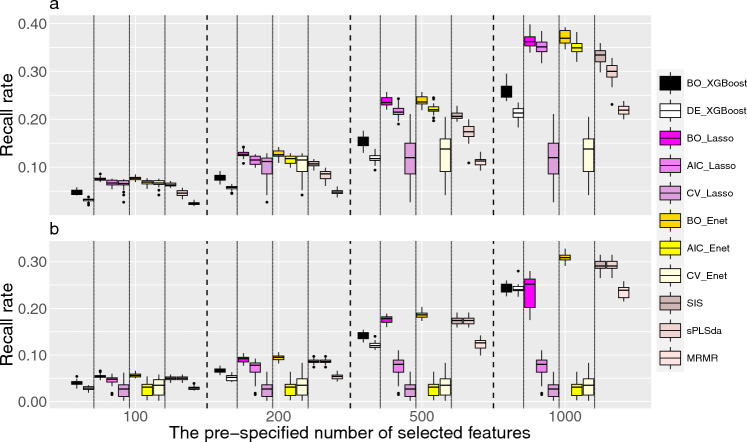
Recall rates for various feature selection methods under linear additive model when the number of causal features is set to 1000. (**a**) Continuous outcomes and (**b**) binary outcomes. The feature selection methods include XGBoost, Lasso, Enet, SIS, sPLSda, and MRMR. The prefixes indicate the method used in hyper-parameter tuning with BO, AIC, CV and DE respectively denoting hyper-parameters selected based on Bayesian optimization, the Akaike Information Criterion, cross-validation, and the default settings in the corresponding R packages.

Figure 1 and Supplementary Figs. S1–S3 depict the recall rates under a linear effect model for both continuous and binary outcomes when the number of causal feature is set to be 1000, 500, 200 and 100, respectively. We found that the recall rates of Bayesian-optimized methods always outperform their corresponding counterparts, and the benefits of Bayesian optimization are more profound when the pre-specified number of features is large. For example, as shown in Fig. 1, BO_Lasso increases the recall rate by 2% when the pre-specified number of selected feature is 100, and this increases to 25% when the pre-specified number of selected feature is 1000. As the pre-specified number of selected features increases, all methods show an upward trend in recall rates. Note that for both Lasso and Enet with hyper-parameters determined by AIC and CV, their recall rate do not change when the pre-specified number of features exceeds the number of features identified according to AIC/CV criterion. With regards to the performance of different feature selection methods, we found that both Lasso and Enet often exhibit better performance, followed by SIS and sPLSda. XGBoost and MRMR have the worse feature selection performance under the linear model. This is mainly because the outcomes are simulated under a linear effect model, which is quite consistent with the assumptions of Lasso and Enet. For binary outcomes, the trends are mostly similar to those of continuous outcomes, where Bayesian optimization showed better performance than their counterparts. However, we noticed that for XGBoost, when the desired number of selected features is large (i.e., 1000), the improvement from Bayesian optimization is limited. This may due to the fact that XGBoost have a relatively large number of hyper-parameters to optimize, and simultaneously identify the subspaces of these hyper-parameters can be challenging, leading to a sub-optimal hyper-parameter settings. Nevertheless, Bayesian optimization always has better or comparable recall rate as compared to their counterparts when outcomes are generated under a linear additive model, indicating its capacity in optimizing the configurations of hyper-parameters.

**Figure 2:**
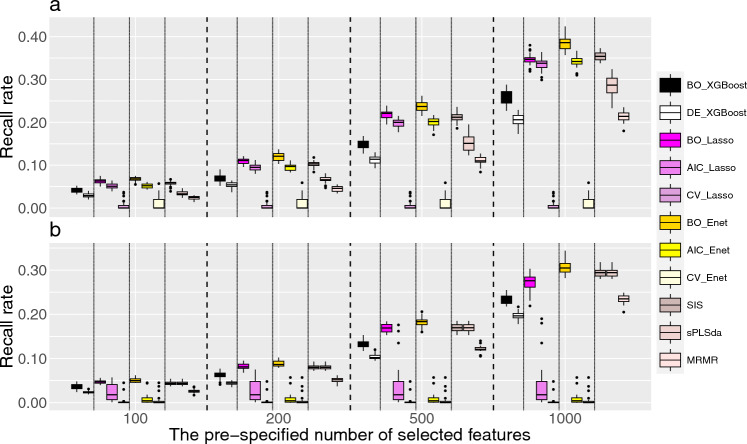
Recall rates for various feature selection methods under non-linear additive model when the number of causal features is set to 1000. (**a**) Continuous outcomes and (**b**) binary outcomes. The feature selection methods include XGBoost, Lasso, Enet, SIS, sPLSda, and MRMR. The prefixes indicate the method used in hyper-parameter tuning with BO, AIC, CV and DE respectively denoting hyper-parameters selected based on Bayesian optimization, the Akaike Information Criterion, cross-validation, and the default settings in the corresponding R packages.

Figure 2 and Supplementary Figs. S4–S6 depict the recall rates under a non-linear effect model for both continuous and binary outcomes when the number of causal feature is set to be 1000, 500, 200 and 100, respectively. The process for non-linear models are similar to those shown in the linear additive models, and the recall rates achieved with Bayesian optimization consistently outperform their corresponding counterparts. It's worth noting that in non-linear models, the recall rates from Lasso and Enet when hyper-parameters were chosen according to AIC or CV can be substantially worse than the other feature selection methods, especially for binary outcomes. However, their Bayesian-optimized counterparts can achieve much better performance. For example, with 1000 features selected, the mean recall rates for Bayesian-optimized Lasso and Enet can respectively reach 35% and 38% for continuous outcomes, whereas the recall rates for CV with Lasso and Enet are less than 5%. We noticed that Lasso and Enet with their default settings generally have worse performance as compared to the other variable selection methods, which is likely due to their underlying modeling assumptions.

### Results from the analysis of gene expression data

**Figure 3:**
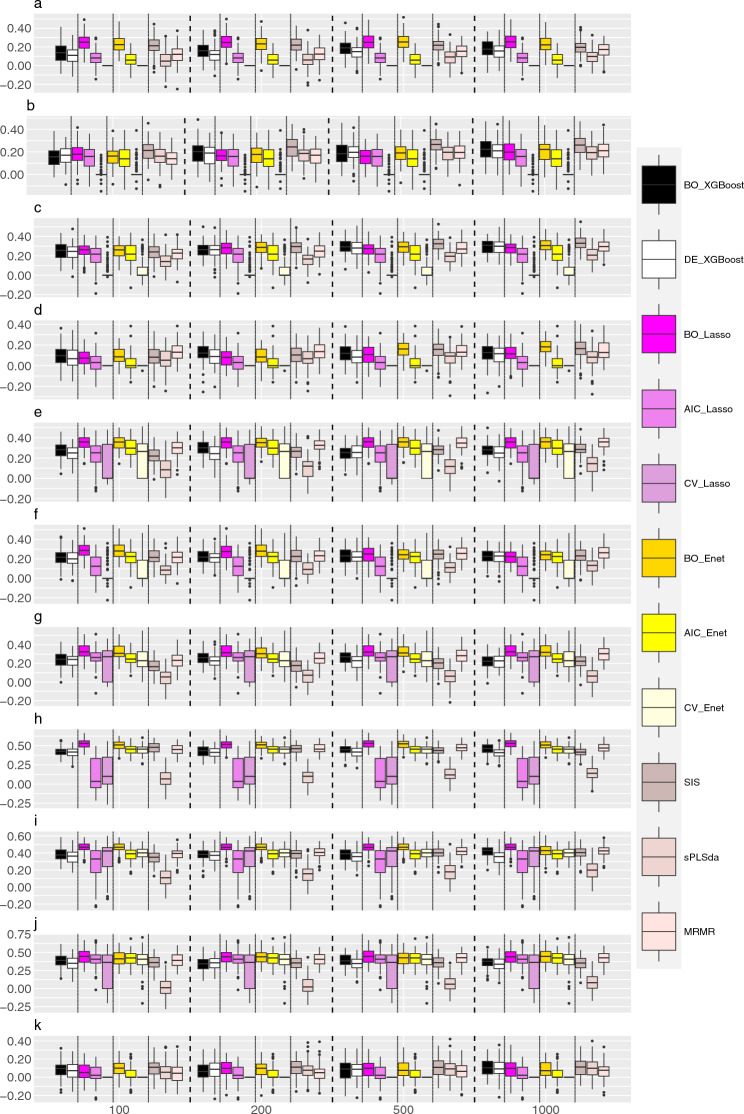
Pearson correlation coefficients for various AD related phenotypes. The features are selected using XGBoost, Lasso, Enet, SIS, sPLSda, and MRMR. The prefixes indicate the method used in hyper-parameter tuning with BO, AIC, CV and DE respectively denoting hyper-parameters selected based on Bayesian optimization, the Akaike Information Criterion, cross-validation, and the default settings in the corresponding R packages. The selected features are further used for building prediction models, where SVM is used. (**a**) hippocampus, (**b**) accumbens, (**c**) amygdala, (**d**) caudate, (**e**) pallidum, (**f**) putamen, (**g**) thalamus, (**h**) gray matter, (**i**) white matter, (**j**) brainstem  + 4th ventricle, and (**k**) white matter hyperintensity.

Figure 3 and Supplementary Figs. S7–S9 display the Pearson correlation coefficients obtained through predictive modeling with SVM, Lasso, RF, and GBM using various feature selection methods on gene expression data from ADNI across different phenotypes, respectively.

Subcortical volumes of AD patients (including the hippocampus, accumbens, amygdala, caudate, pallidum, putamen, and thalamus) typically undergo atrophy, which is associated with memory loss and other cognitive impairments. For instance, AD patients often exhibit a reduction in hippocampal volume, as the hippocampus is one of the earliest brain regions affected by AD^45^. In our analyses, we noticed that the gene expression levels have different predictive power on these subcortical volumes related phenotypes. For example, when utilizing Bayesian-optimized Lasso and Enet as feature selection methods and employing SVM as the predictive model, the predictive accuracy for pallidum reaches its peak, with an average Pearson coefficient of 0.38 (Fig. 3e). However, for the prediction of caudate, the optimal combination involves using SIS as the feature selection method and RF as the predictive model, and this model resulted in an average Pearson coefficient of 0.2 (Supplementary Fig. S8), which is substantially lower than that from the prediction of pallidum.

Changes in the volumes of gray matter, white matter, and the brainstem + 4th ventricle observed in T1 structural brain MRI can serve as indicators of brain atrophy and the progression of neurodegenerative changes in AD, which may be associated with a decline in brain function^46^. The gene expression levels can have a moderate predictive power on these traits, with most methods achieving an average Pearson coefficient of around 0.4. Specifically, within the gray matter, the best predictive performance is attained when using Bayesian-optimized Lasso as the feature selection method and SVM as the predictive model, resulting in an average Pearson coefficient of 0.55 (Fig. 3h).

Changes in the volume of white matter hyperintensities from T2-weighted brain MRI typically represent white matter damage or degenerative alterations. In AD, these regions may enlarge, potentially reflecting the disease's impact on the microstructure of the brain^47^. In our analyses, we found that gene expression levels lack the capacity in predicting them, regardless of the methods used. The optimal combination is using SIS as the feature selection method and RF as the predictive model, yielding an average Pearson coefficient of 0.16 (Supplementary Fig. S8).

Overall, we have observed that features pre-selected through Bayesian optimization generally exhibit better predictive accuracy compared to their respective counterparts. For example, as illustrated in Fig. 3a, for hippocampus, when employing Bayesian-optimized Lasso as the feature selection method, the model achieves an average Pearson correlation coefficient of 0.25. In contrast, the Pearson correlation coefficients for the Lasso models with AIC and CV are 0.08 and 0, respectively. It is worth noting there are some exceptions where Bayesian-optimized method has similar performance as its original method (e.g., the brainstem  + 4th ventricle). Furthermore, the advantages shown by Bayesian optimization become more pronounced when constructing the final predictive model using SVM. For instance, in the predictions of pallidum and putamen, the Bayesian-optimized Enet model demonstrates a notably pronounced advantage over the AIC-based Enet model (Fig. 3e,f). However, when utilizing other prediction models (i.e., Lasso, RF, and GBM), the Bayesian-optimized models can not exhibit a significant advantage (Supplementary Figs. S7–S9).

## Discussion

Bayesian optimization efficiently explores subspaces of hyper-parameters that are likely to result in optimal solutions, and it can substantially improve the performance of machine learning models where hyper-parameter tuning is involved^48^. Existing Bayesian optimization mainly focuses on prediction performance of the models, and it remains unknown for its impact on feature selection, which is of critical importance for the analysis of high-dimensional data. Similar to prediction models, the performance of many existing feature selection methods relies on the choice of their associated hyper-parameters, where their fine-tuning can be challenging^49^. In this project, we systematically investigated the impact of Bayesian optimization on feature selection algorithms that have hyper-parameters. Through simulation studies, we found that Bayesian optimization can improve the recall rate for a given pre-specified number of selected features. Through the prediction analysis of various AD-related phenotypes, we found that prediction models built with gene expression levels that are selected by Bayesian-optimized feature selection methods tend to have better prediction accuracy.

In our simulation studies when the true outcome-related features are known, we used the recall rate to assess the impact of Bayesian optimization on feature selection. We noticed that Bayesian optimization generally help to improve recall rate, and the selection features have better overlap with causal features when compared to their default settings. For example, the average recall rate for Lasso with the tuning parameter determined based on cross-validation is 12%, whereas the Bayesian-optimized one can reach 35%. While Bayesian-optimized feature selection methods consistently outperform their counterparts, the relative advantages depend on the complexity of the hyper-parameter spaces. For Lasso where only one parameter needs to be optimized, the subspace of hyper-parameters that is likely to result in the best performance of the objective function is relatively easy to identify. Therefore, Bayesian-optimized lasso usually has much better performance than those non-optimized ones. However, for XGBoost, it involves multiple hyper-parameters such as ‘Learning rate’, ‘Subsample’ and ‘penalty term’, the subspaces of these parameters that lead to the optimal performance of the objective function can be hard to determine, and the relative advantage of Bayesian optimization over the traditional method can vary. Bayesian optimization determines the best configurations of hyper-parameters via iteratively estimating the posterior distribution of the objective function and the subspaces of the hyper-parameters that are likely to achieve the optimized objective function. Therefore, the number of iterations adopted by Bayesian optimization can also affect its performance. For complex hyper-parameter spaces, limited number of iterations may fail to identify the subspaces where the hyper-parameters should lay on, and thus can provide a setting that performs worse than the default settings. On contrary, an excessive number of iterations can lead to heavy computation, especially for methods that are computationally intensive by themselves. This is mainly because estimating the posterior distribution of the objective function requires repeatedly solving the original problem. Therefore, in practice, we recommend to consider the complexity of the hyper-parameter space and make a trade-off between the computational cost and optimality of the hyper-parameters.

The significance of predicting AD lies in its ability to offer opportunities for early intervention, aiding patients and their families in planning for the future, providing patients with the chance to participate in clinical trials, and enhancing their quality of life through symptom management and alleviation^50^. All of these contribute to slowing the progression of the disease and enhancing the overall quality of life for patients. Gene expression data has provided crucial information for elucidating the pathogenic mechanisms, drug development, precise diagnosis, and early prediction of AD by revealing genetic variations, risk genes, and associated molecular pathways^51^. In this research, we used various feature selection methods, including the Bayesian-optimized methods, to perform feature selection. We then used commonly used machine learning methods to predict various AD-related phenotypes using the pre-selected features. We found that gene expression has various levels of predictive power on AD-related traits. For example, for the subcortical volumes related traits, the best prediction model for prediction pallidum can reach an average Pearson correlation of 0.38, whereas the best model for caudate only obtained an average Pearson correlation of 0.2. This suggests that the transcriptomics do not affect AD-related traits in the same fashion. For example, ‘*APOE4*’ regulates the expression levels of β-amyloid plaques, tau protein, and TDP43 protein in the brains of AD patients, clues have been discovered in the pallidum region^52^. Furthermore, pathogenic mutations in ‘*APP*’ lead to excessive production of Aβ amyloid-like proteins, ultimately resulting in AD, which is reflected in the hippocampus^53^. Structural brain MRI is often used in the diagnosis and treatment of AD, and it can provide valuable information on the pathological changes of the brain functions^54^. We noticed that gene expression levels tends to predict moderately well for the information observed from T1 structural brain MRI scan, and the average Pearson correlations for these traits reached 0.4. Changes in the volumes of gray matter, white matter and the brainstem  + 4th ventricle reflected by the T1 structural brain MRI represents the neurodegenerative changes in AD and gene expression levels have been shown to be associated with these measures. For example, research has indicated that the gene ‘*SLC2A3*’ is associated with AD, and this is reflected in the gray matter phenotype^55^. Interestingly, we found that gene expression generally cannot predict white matter hyperintensity that is measured by T2-weighted brain MRI well. Our best model only achieved an average Pearson correlation of 0.16. White matter hyperintensity is often associated with vascular abnormalities^56^. Although vascular abnormalities are associated with AD^57^, our study has found that gene expression levels can not reflect the vascular abnormalities and it is likely that these two factors affect AD in a different pathways. It is worth noting that for most of the AD-related phenotypes, features selected by Bayesian optimization and the prediction models built with SVM achieved the best prediction performance.

Our study has some limitations. First, during the feature selection process, we followed the mainstream of the existing features selection methods and mainly focused on the main effects. We completely ignore the potential interactions. For high-dimensional molecular data, epistasis widely exists^58^. It could be of interest to investigate the impact of Bayesian optimization when interactions are further considered. Second, we have used gene expression data to build prediction models for various AD-related phenotypes and calculated the prediction accuracy based on the cross-validation using ADNI. Future studies are needed to further validate the prediction models using external datasets. Nevertheless, our study has provided sufficient evidence to suggest that Bayesian optimization can benefit feature selection and enhance the performance of downstream tasks.

In summary, Bayesian optimization can enhance the performance of feature selection methods, which can greatly facilitate downstream tasks such as disease risk prediction. It is worth noting that the complexity of the objective function for Bayesian optimization as well as the complexity of the hyper-parameter spaces can have major impact on the performance of Bayesian optimization. We recommend that trade-off should be made between the computational cost and optimality of the hyper-parameters in practice.

### Supplementary Information

Supplementary Information.

## Data Availability

The ADNI datasets can be found at http://adni.loni.ucla.edu/.
