# Supplementary material for: The impact of Bayesian optimization on feature selection
Source: Sci Rep. 2024 Feb 17;14:3948. doi: 10.1038/s41598-024-54515-w (PMC10873405; doi:10.1038/s41598-024-54515-w)
Supplement: Supplementary file 1 — Supplementary Information. [file 41598_2024_54515_MOESM1_ESM.pdf]

Supplementary Material

The impact of Bayesian optimization on feature selection

Kaixin Yang, Long Liu, Yalu Wen

Supplementary Figure S1 to S9

Supplementary Table S1 to S2

1. Supplementary Figures

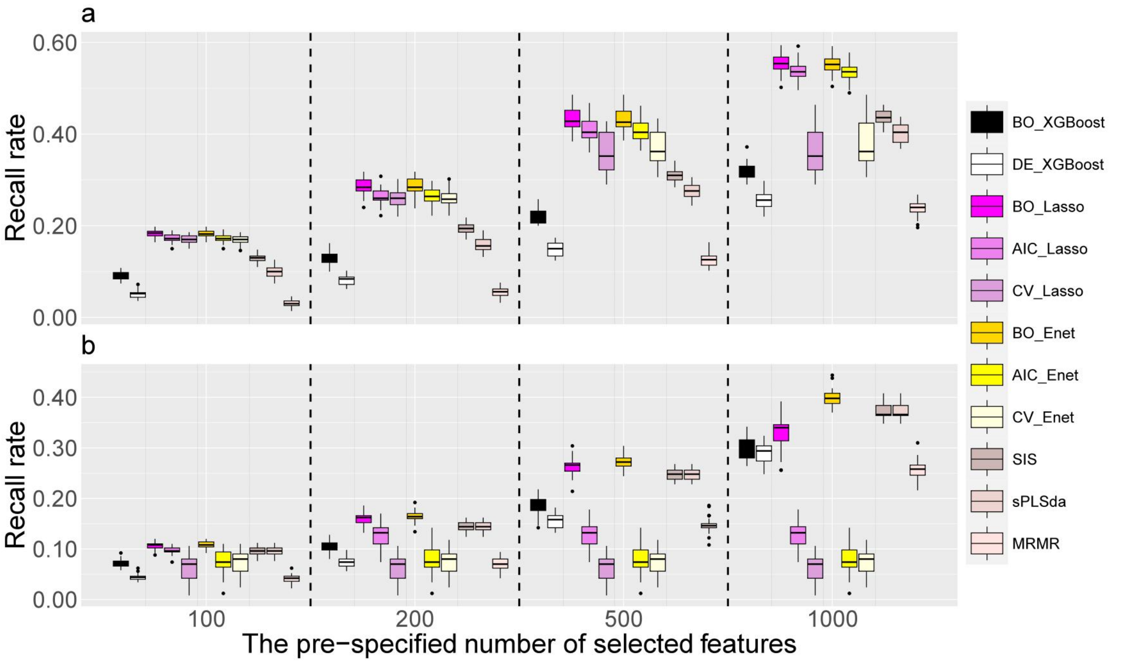

Supplementary Figure S1. Recall rates for various feature selection methods under linear additive model when the number of causal features is set to 500. (**a**) Continuous outcomes and (**b**) binary outcomes. The feature selection methods include XGBoost, Lasso, Enet, SIS, sPLSda, and MRMR. The prefixes indicate the method used in hyper-parameter tuning with BO, AIC, CV and DE respectively denoting hyper-parameters selected based on Bayesian optimization, the Akaike Information Criterion, cross-validation, and the default settings in the corresponding R packages.

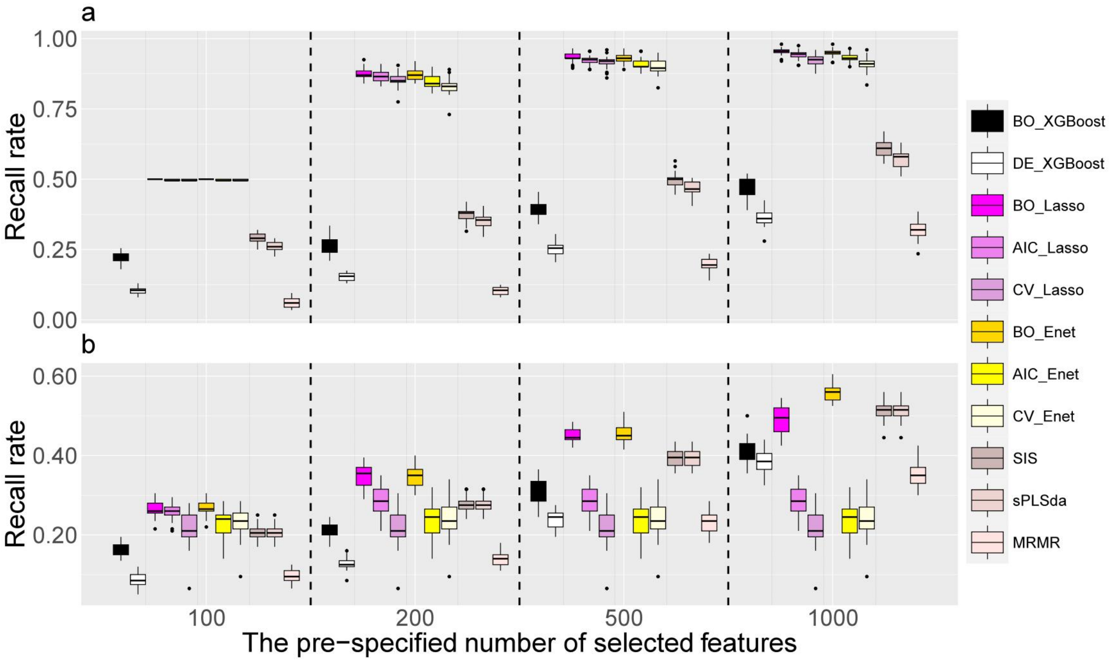

Supplementary Figure S2. Recall rates for various feature selection methods under linear additive model when the number of causal features is set to 200. (**a**) Continuous outcomes and (**b**) binary outcomes. The feature selection methods include XGBoost, Lasso, Enet, SIS, sPLSda, and MRMR. The prefixes indicate the method used in hyper-parameter tuning with BO, AIC, CV and DE respectively denoting hyper-parameters selected based on Bayesian optimization, the Akaike Information Criterion, cross-validation, and the default settings in the corresponding R packages.

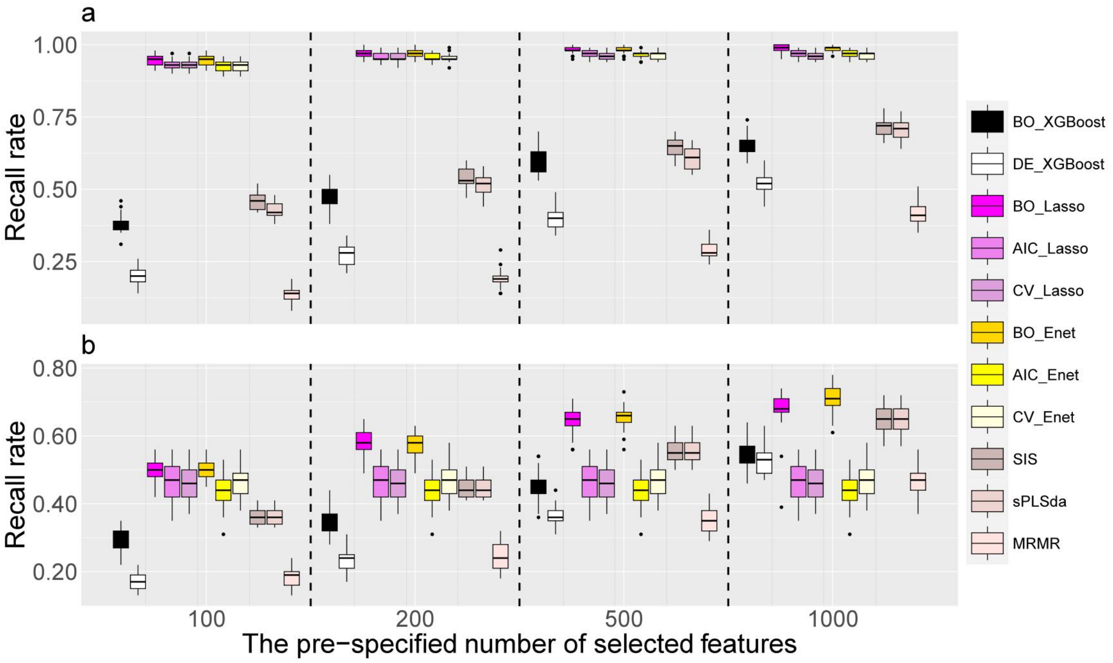

Supplementary Figure S3. Recall rates for various feature selection methods under linear additive model when the number of causal features is set to 100. (**a**) Continuous outcomes and (**b**) binary outcomes. The feature selection methods include XGBoost, Lasso, Enet, SIS, sPLSda, and MRMR. The prefixes indicate the method used in hyper-parameter tuning with BO, AIC, CV and DE respectively denoting hyper-parameters selected based on Bayesian optimization, the Akaike Information Criterion, cross-validation, and the default settings in the corresponding R packages.

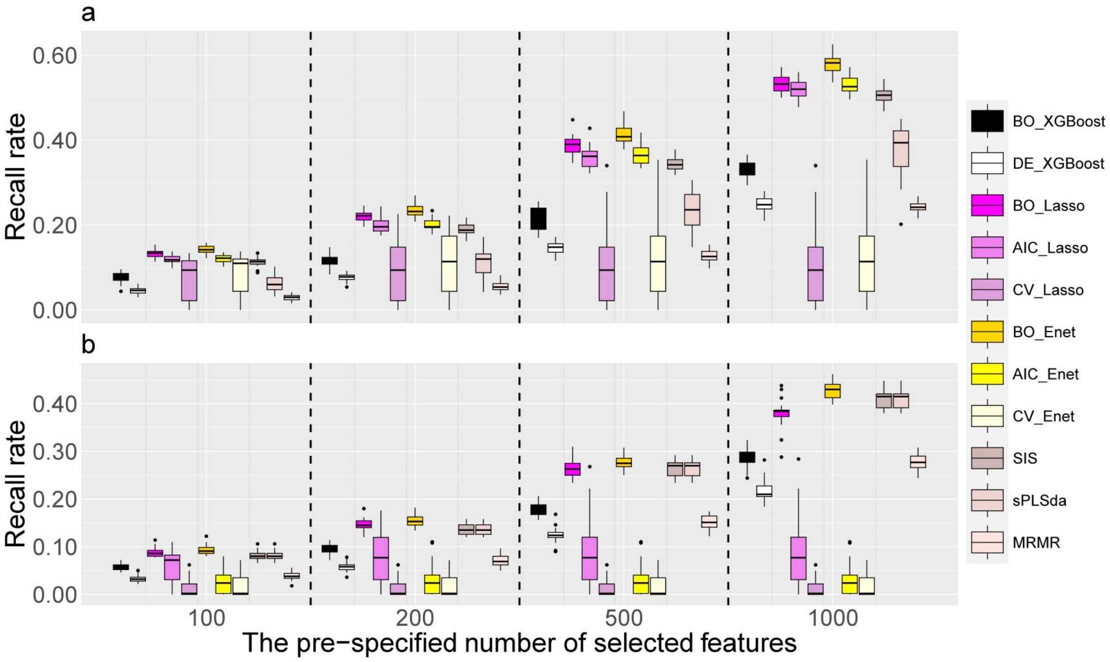

Supplementary Figure S4. Recall rates for various feature selection methods under non-linear additive model when the number of causal features is set to 500. (**a**) Continuous outcomes and (**b**) binary outcomes. The feature selection methods include XGBoost, Lasso, Enet, SIS, sPLSda, and MRMR. The prefixes indicate the method used in hyper-parameter tuning with BO, AIC, CV and DE respectively denoting hyper-parameters selected based on Bayesian optimization, the Akaike Information Criterion, cross-validation, and the default settings in the corresponding R packages.

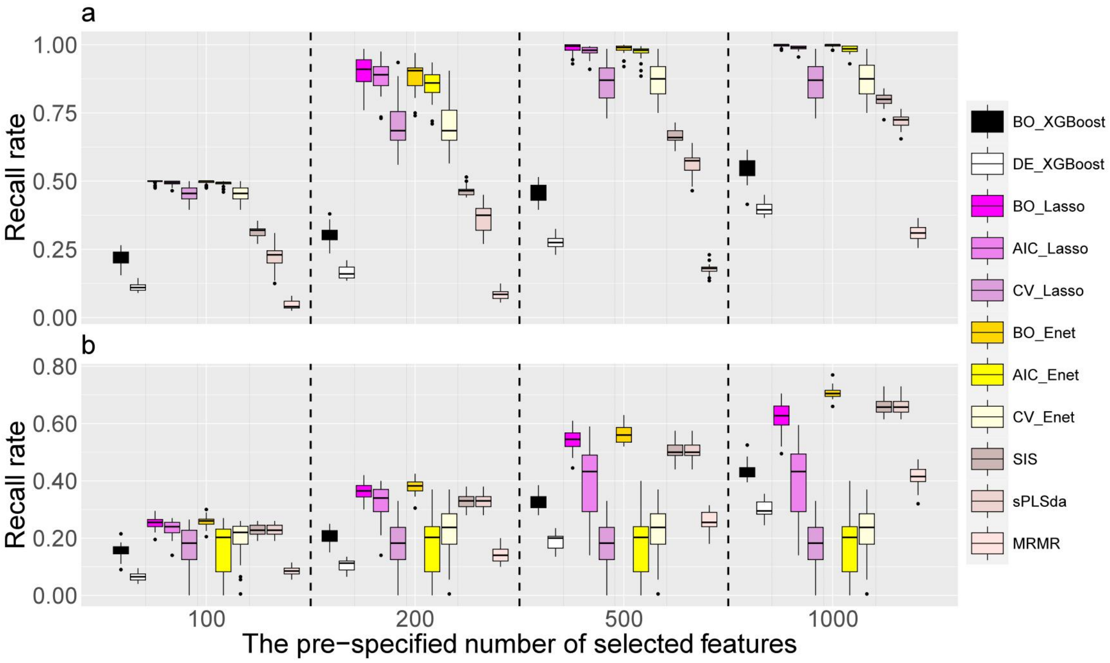

Supplementary Figure S5. Recall rates for various feature selection methods under non-linear additive model when the number of causal features is set to 200. (**a**) Continuous outcomes and (**b**) binary outcomes. The feature selection methods include XGBoost, Lasso, Enet, SIS, sPLSda, and MRMR. The prefixes indicate the method used in hyper-parameter tuning with BO, AIC, CV and DE respectively denoting hyper-parameters selected based on Bayesian optimization, the Akaike Information Criterion, cross-validation, and the default settings in the corresponding R packages.

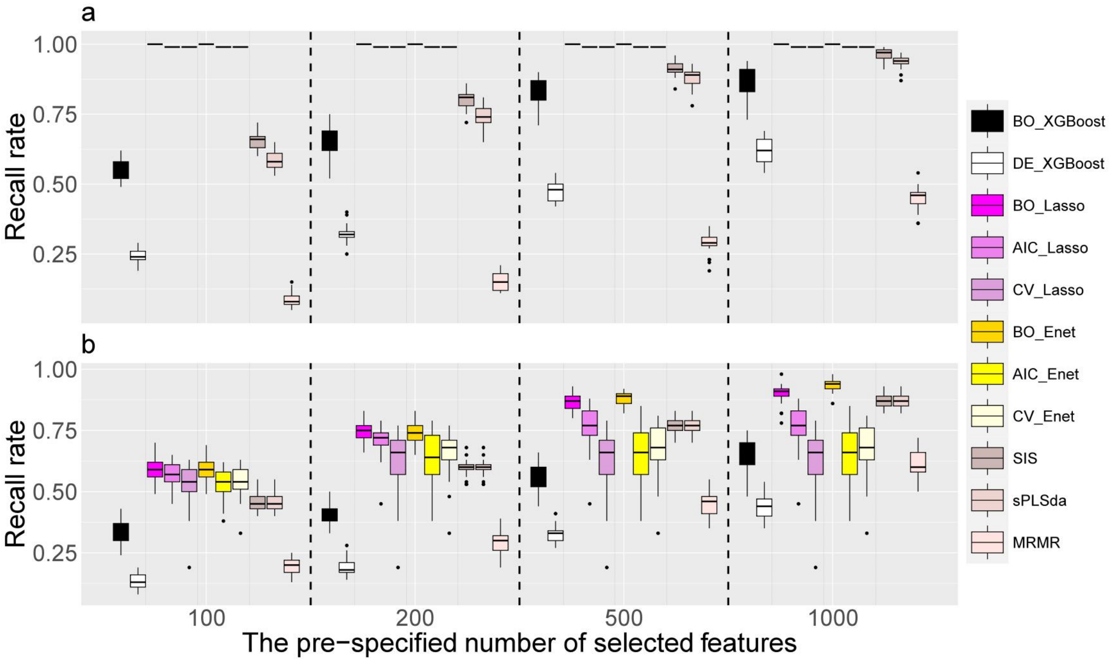

Supplementary Figure S6. Recall rates for various feature selection methods under non-linear additive model when the number of causal features is set to 100. (**a**) Continuous outcomes and (**b**) binary outcomes. The feature selection methods include XGBoost, Lasso, Enet, SIS, sPLSda, and MRMR. The prefixes indicate the method used in hyper-parameter tuning with BO, AIC, CV and DE respectively denoting hyper-parameters selected based on Bayesian optimization, the Akaike Information Criterion, cross-validation, and the default settings in the corresponding R packages.

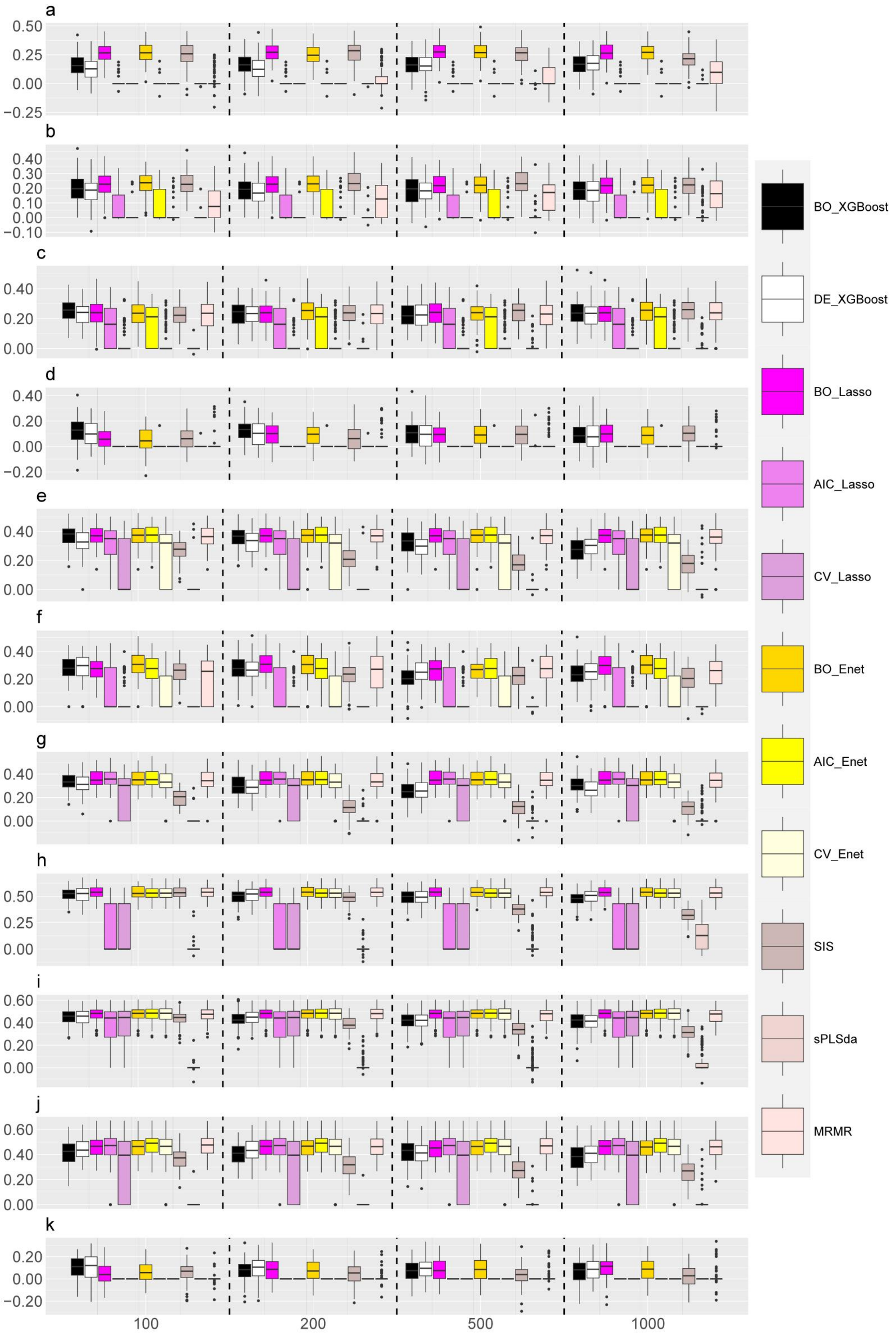

Supplementary Figure S7. Pearson correlation coefficients for various AD related phenotypes. The features are selected using XGBoost, Lasso, Enet, SIS, sPLSda, and MRMR. The prefixes indicate the method used in hyper-parameter tuning with BO, AIC, CV and DE respectively denoting hyper-parameters selected based on Bayesian optimization, the Akaike Information Criterion, cross-validation, and the default settings in the corresponding R packages. The selected features are further used for building prediction models, where Lasso is used. (**a**) hippocampus, (**b**) accumbens, (**c**) amygdala, (**d**) caudate, (**e**) pallidum, (**f**) putamen, (**g**) thalamus, (**h**) gray matter, (**i**) white matter, (**j**) brainstem+4th ventricle, and (**k**) white matter hyperintensity.

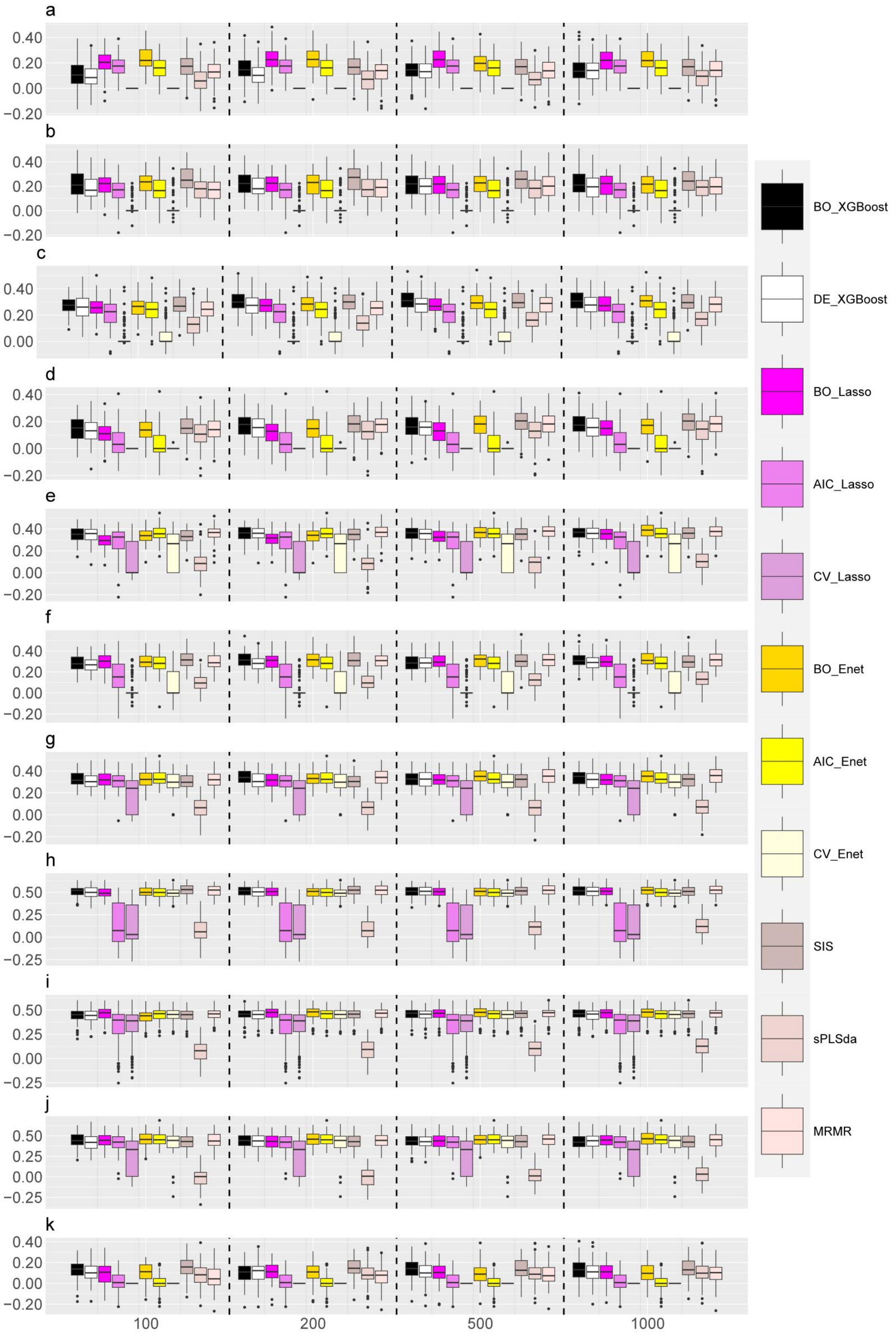

Supplementary Figure S8. Pearson correlation coefficients for various AD related phenotypes. The features are selected using XGBoost, Lasso, Enet, SIS, sPLSda, and MRMR. The prefixes indicate the method used in hyper-parameter tuning with BO, AIC, CV and DE respectively denoting hyper-parameters selected based on Bayesian optimization, the Akaike Information Criterion, cross-validation, and the default settings in the corresponding R packages. The selected features are further used for building prediction models, where RF is used. (**a**) hippocampus, (**b**) accumbens, (**c**) amygdala, (**d**) caudate, (**e**) pallidum, (**f**) putamen, (**g**) thalamus, (**h**) gray matter, (**i**) white matter, (**j**) brainstem+4th ventricle, and (**k**) white matter hyperintensity.

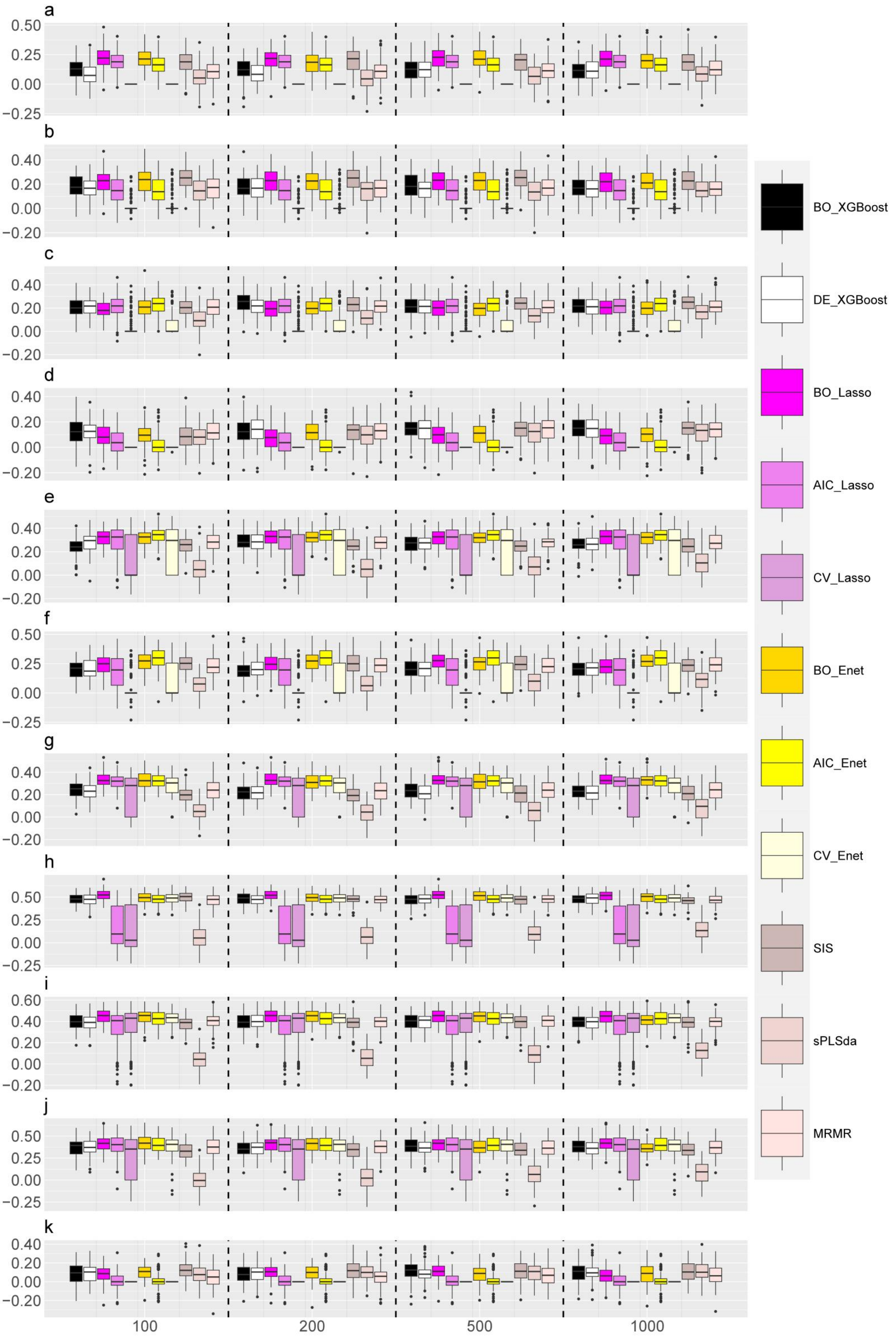

Supplementary Figure S9. Pearson correlation coefficients for various AD related phenotypes. The features are selected using XGBoost, Lasso, Enet, SIS, sPLSda, and MRMR. The prefixes indicate the method used in hyper-parameter tuning with BO, AIC, CV and DE respectively denoting hyper-parameters selected based on Bayesian optimization, the Akaike Information Criterion, cross-validation, and the default settings in the corresponding R packages. The selected features are further used for building prediction models, where GBM is used. (**a**) hippocampus, (**b**) accumbens, (**c**) amygdala, (**d**) caudate, (**e**) pallidum, (**f**) putamen, (**g**) thalamus, (**h**) gray matter, (**i**) white matter, (**j**) brainstem+4th ventricle, and (**k**) white matter hyperintensity.

2. Supplementary Table

Supplementary Table S1. Hyper-parameters in XGBoost.

Hyper-parameter	Optimization ranges	Default value
Learning rate	(0.001, 0.3)	0.3
Gamma[a]	(0, 1)	0
Max Depth[b]	(3, 15)	6
Min Child Weight[c]	(1, 7)	1
Subsample[d]	(0.5, 1)	1
Colsample Bytree[e]	(0, 1)	1
L_2 penalty term	(0, 1)	1
L_1 penalty term	(0, 1)	0

a: It specifies minimum loss reduction required to make a further partition on a leaf node of the tree.
b: It defines the maximum depth of a tree.
c: It sets the minimum sum of instance weight (Hessian) needed in a child.
d: It specifies the fraction of samples used for growing trees during training.
e: It's the fraction of features used when constructing each tree.

Supplementary Table S2. The demographic details of gene expression data in the Alzheimer's Disease Neuroimaging Initiative study.

Demographics	Description	Number	Percentage
Age	55 - 59.9	24	3.2
	60 - 69.9	199	26.7
	70 - 79.9	391	52.6
	80 - 89.9	127	17.1
	90 - Above	3	0.4
	Total	744	100.0
Gender	Male	408	54.8
	Female	336	45.2
	Total	744	100.0
Education	6 - 12	108	14.5
	13 - 16	315	42.3
	17 - Above	321	43.2
	Total	744	100.0
Ethnicity	Hisp/Latino	17	2.3
	Not Hisp/Latino	724	97.3
	Unknown	3	0.4
	Total	744	100.0
Race	Am Indian/Alaskan	2	0.3

	Asian	11	1.5
	Black	30	4.0
	Hawaiian/Other PI	2	0.3
	More than one	7	0.9
	Unknown	2	0.3
	White	690	92.7
	Total	744	100.0
Marital	Divorced	71	9.5
	Married	563	75.7
	Never married	19	2.6
	Unknown	3	0.4
	Widowed	88	11.8
	Total	744	100.0
